# Plant Growth Promotion by Volatile Organic Compounds Produced by *Bacillus subtilis* SYST2

**DOI:** 10.3389/fmicb.2017.00171

**Published:** 2017-02-07

**Authors:** Hafiz A. S. Tahir, Qin Gu, Huijun Wu, Waseem Raza, Alwina Hanif, Liming Wu, Massawe V. Colman, Xuewen Gao

**Affiliations:** ^1^Department of Plant Pathology, College of Plant Protection, Nanjing Agricultural University, Key Laboratory of Integrated Management of Diseases and Pests, Ministry of EducationNanjing, China; ^2^Jiangsu Key Lab for Organic Solid Waste Utilization, Nanjing Agricultural UniversityNanjing, China

**Keywords:** bacterial VOCs, *Bacillus subtilis* SYST2, growth promotion, phytohormones, albuterol, 1, 3-propanediole

## Abstract

Bacterial volatiles play a significant role in promoting plant growth by regulating the synthesis or metabolism of phytohormones. *In vitro* and growth chamber experiments were conducted to investigate the effect of volatile organic compounds (VOCs) produced by the plant growth promoting rhizobacterium *Bacillus subtilis* strain SYST2 on hormone regulation and growth promotion in tomato plants. We observed a significant increase in plant biomass under both experimental conditions; we observed an increase in photosynthesis and in the endogenous contents of gibberellin, auxin, and cytokinin, while a decrease in ethylene levels was noted. VOCs emitted by SYST2 were identified through gas chromatography-mass spectrometry analysis. Of 11 VOCs tested in glass jars containing plants in test tubes, only two, albuterol and 1,3-propanediole, were found to promote plant growth. Furthermore, tomato plants showed differential expression of genes involved in auxin (*SlIAA1. SlIAA3*), gibberellin (*GA20ox-1*), cytokinin (*SlCKX1*), expansin (*Exp2, Exp9. Exp 18*), and ethylene (*ACO1*) biosynthesis or metabolism in roots and leaves in response to *B. subtilis* SYST2 VOCs. Our findings suggest that SYST2-derived VOCs promote plant growth by triggering growth hormone activity, and provide new insights into the mechanism of plant growth promotion by bacterial VOCs.

## Introduction

Volatile organic compounds (VOCs) produced by plant growth promoting rhizobacterium (PGPR) have been proved to have the potential to control plant pathogens, to stimulate plant growth, and to induce systemic disease resistance (Ryu et al., 2003; Lee et al., 2012; Park et al., 2015; Raza et al., 2016; Tahir et al., 2017). PGPRs, or products derived by PGPRs, usually require physical contact with plant parts for the stimulation of plant growth (Xie et al., 2014). However, many types of bacteria can regulate plant growth from a distance without any contact, suggesting the possibility that these bacteria emit invisible volatile compounds that promote or inhibit plant growth. Nearly 350 bacterial species have been reported to produce around 846 different potential VOCs, with 5431 synonyms (Lemfack et al., 2014).

Bacteria having potential growth-promoting activity include species of *Pseudomonas, Bacillus, Stenotrophomonas, Serratia*, and *Arthrobacter* (Ryu et al., 2003; Bailly and Weisskopf, 2012; Kai and Piechulla, 2014; Park et al., 2015; Raza et al., 2016). Ryu et al. (2003) first reported two VOCs (2,3-butanediol and acetoin) that triggered growth promotion. *Bacillus megaterium* XTBG34 produces 2-pentylfuran which promoted the growth of *Arabidopsis thaliana* plants by two fold after 15 days of treatment (Zou et al., 2010). More recently, Park et al. (2015) reported that 13-tetradecadien-1-ol, 2-butanone, and 2-methyl-n-1-tridecene, produced by *Pseudomonas fluorescens* SS101, enhanced the growth of *Nicotiana tabacum*.

Plant growth promoting rhizobacteria have been reported to promote plant growth by regulating plant hormones, such as auxins, gibberellins, cytokinins, and ethylene (Timmusk et al., 1999; Glick, 2005; Bailly and Weisskopf, 2012; Xie et al., 2014), and by increasing the availability of N, P, and Fe in the soil (Kim et al., 2011; Ortíz-Castro et al., 2014). However, studies on interactions between VOCs produced by PGPR and plant hormones involved in growth processes are at the preliminary stages. Over the past decade, studies have shown that bacterial VOCs can also interact with these phytohormones by taking part in morphogenetic processes, resulting in the stimulation of plant growth (Ryu et al., 2003; Zhang et al., 2007; Ortíz-Castro et al., 2014). In a transcriptional analysis of *Arabidopsis*, auxin regulation by VOCs from *Bacillus subtilis* GBO3 resulted in the initiation of growth promotion. Auxin accumulation decreased in leaves while it increased in roots after exposure to VOCs, possibly due to the triggering of basipetal auxin transport (Zhang et al., 2007). VOCs altered the transcription of genes related to ethylene biosynthesis; *ACO2. ACS4. ACS12*, and *SAM-2*, and also genes involved in the ethylene response; *CHIB. ERF1*, and *GST* (Kwon et al., 2010). An increase in photosynthesis and chlorophyll content was observed in *Arabidopsis* seedlings exposed to VOCs from *B. subtilis* GBO3 (Xie et al., 2009). *Bacillus* species are considered to be the most effectivebecause they have the capability to produce spores that can survive even in adverse environmental conditions (Francis et al., 2010).

The main objective of this study was to explore the growth promoting activity of VOCs produced by *B. subtilis* SYST2 in tissue culture jars and in pots by evaluating the effect of these VOCs on photosynthesis, gibberellin, cytokinin, indole 3-acetic acid, and ethylene content in tomato plants. VOCs emitted by SYST2 increased plant biomass as compared to the water control and *E. coli* DH5α (a non-VOC producer) both in sealed tissue culture jars and pots. Albuterol and 1,3-propanediole caused enhanced growth in plants exposed to different concentrations. A significant increase in photosynthetic activity was observed along with an increase in gibberellin and auxin contents and a decrease in ethylene content in tomato plants when exposed to VOCs. In agreement with these observations, VOCs altered the relative expression levels of genes relating to expansin (*Exp2. Exp9. Exp18*), auxin (*SlIAA1. SlIAA3*), gibberellin (*GA20ox-1*), and ethylene biosynthesis (*ACO1*).

## Materials and Methods

### Bacterial Strain, Plant Material, and Plant Growth Conditions

*Bacillus subtilis* strain SYST2, which was previously isolated by our lab (Laboratory of Biocontrol and Bacterial Molecular Biology, Nanjing Agriculture University, Nanjing, China), was used in this study. *B. subtilis* SYST2 was grown on Luria-Bertani (LB) medium at 37°C overnight, and stock cultures were stored in LB broth supplemented with 30% glycerol at -20°C. Seeds of tomato (*Solanum lycopersicum* L.) were surface-sterilized by soaking in 70% ethanol for 1 min followed by soaking in 50% sodium hypochlorite for 15 min, after that seeds were rinsed 3–4X in sterile distilled water. The sterilized seeds were placed in test tubes containing 0.5X Murashige and Skoog (MS) salt medium (Murashige and Skoog, 1962; pH 5.7) supplemented with 1.5% sucrose and solidified with 0.8% agar.

### Growth-Promoting Activity of SYST2 VOCs in Tissue Culture Jars and in Pot Experiments

Four test tubes containing 0.5X MS medium were placed in tissue culture jars (12 cm × 7 cm). A Petri plate (4 cm × 1.5 cm) containing MS media for SYST2 growth was placed in the bottom of each jar. One surface-sterilized tomato seed was placed in each test tube, and 20 μl of SYST2 culture (10^7^ CFU/ml) was then dropped on a sterilized filter paper disk on the small plate. The jars were closed with a lid, sealed completely with Parafilm, and placed in a plant growth chamber at 25°C for 14 days under a photoperiod of 12 h light/12 h dark. The effect of VOCs produced by SYST2 on plant growth was determined by measuring the differences in fresh green weights, and by measuring the root and shoot lengths of the tomato seedlings. For the pot experiments, SYST2 was inoculated on a Petri plate placed at the bottom of a tissue culture jar (12 cm × 10 cm) as described by Park et al. (2015). Three germinated tomato seedlings of equal size were transferred to the plastic pots (6 cm × 3 cm) containing soil (appropriate amounts of sand, clay, and organic matter), fixed on the glass jars and sealed with Parafilm to avoid the escape of VOCs produced by SYST2. Five or six small holes (2 mm) were made in the bottom of the pots to allow the roots to be exposed to the VOCs. The pots fixed on the jars were then placed at 25°C under a 12 h light/12 h dark photoperiod for 30 days. Plant growth promoting activity was determined by measuring the differences in root and shoot lengths, fresh green weight, and leaf area between the controls and plants exposed to VOCs.

### GC-MS Profile of VOCs Produced by SYST2

A 20-μl suspension of SYST2 cells was used to inoculate 30 ml of MS agar medium in a 100-ml vial at 28°C. Only MS agar medium was used as control. To collect the VOCs, a 2 cm divinyl benzene/carboxen/PDMS (DCP, 50/30 μm) solid phase microextraction (SPME) fiber (Supelco, Bellefonta, PA, USA) was used. After 5 days, the SPME fiber was inserted into the headspace of the vial containing bacteria and incubated at 50°C for 30 min. Gas chromatography-mass spectrometry (GC-MS) analysis was performed using a Bruker 450-GC gas chromatograph in combination with a Bruker 320-MS mass spectrometer as described by Farag et al. (2006). Helium gas was used as the carrier at a flow rate of 1 ml min^-1^. The SPME fibers were desorbed at 220°C for 5 min, and GC–MS was run for 25 min. The starting temperature of the column was 35°C for 3 min, which was increased to 180°C at a rate of 10°C/min, further increased to 240°C at 4°C/min, and then held for 5 min. The mass spectrometer was operated in the electron ionization mode at 70 eV with a source temperature of 220°C, with continuous scanning from 50 to 500 m/z. The mass spectra data for the volatile compounds were analyzed using the data in the NIST/EPA/NIH Mass Spectrum Library. All of the compounds that we identified as being emitted by SYST2 were purchased from Sigma-Aldrich or Aladdin and tested individually for their plant growth promoting ability.

### Effect of SYST2 VOCs on Hormone Profiles and Photosynthesis

Plant samples were collected at 14, 21, and 28 days after exposure to VOCs produced by SYST2. The endogenous contents of gibberellin, auxin, and cytokinin were determined based on Kelen et al. (2004). Fresh samples of 0.5 g were ground in 5 ml 50% methanol, stirred for 10–12 h at 4°C, and centrifuged at 10,000 rpm for 10 min. The extracts were stored at -70°C and the extraction process repeated three times by adding 2 ml of methanol to each extraction. To purify the samples, 0.2 g polyvinyl pyrrolidone (PVP) was added and stirred at shaker for 1 h at 4°C, by adjusting pH to 8.5. The extracts were filtered through C-18 columns at 4°C and dried under vacuum in the cold. The dry samples were dissolved in 5 ml of 50% methanol and mixed thoroughly. The extracted samples were loaded onto a reverse phase HPLC (Shimadzu) C-18 column (250 mm × 4.60 mm, 5 μm) (Kelen et al., 2004). The standards used for the quantification of hormones were purchased from Sigma-Aldrich Corp. (St. Louis, MO, USA). The ethylene contents in the tomato plants were analyzed using a plant ETH ELISA kit (ColorfulGene Bio-Tek, Wuhan, China) after 14, 21, and 28 days of exposure to SYST2-VOCs.

To measure the photosynthesis rate, five maximum light-exposed leaves from three plants were assayed using an open IRGA LI-COR 6400 system (LI-6400, Li-Cor Inc., USA). Net photosynthetic rate (Pn) and stomatal conductance (gs) were noted under light saturated conditions at photosynthetic photon flux density of 1000 mmol photons m^-2^s^-1^ at 25°C and 380 mol mol^-1^ CO_2_ concentration.

### Effect of SYST2 VOCs on Transcription of Genes Involved in Phytohormone Biosynthesis

Total RNA was extracted from leaf samples taken at 7, 14, 21, and 28 days after exposure to SYST2 VOCs using TRIzol reagent (Invitrogen Biotechnology Co., Carlsbad, CA, USA) according to the manufacturer’s instructions. First-strand cDNA was synthesized using reverse transcriptase (TaKaRa Bio Inc., Tokyo, Japan) and random hexamer primers. Real-time PCR was performed with SYBR Green/Fluorescent qPCR master mix (Takara) on a Roche-480 system (Roche) using the *EF-1α* gene (Berberich et al., 1995) as an internal reference.

The relative expression levels of *Exp2, Exp9, Exp18. ACO-1. GA (20ox-1). SlIAA1, SlIAA3*, and *SlCKX1* were assayed. For qRT-PCR, the instrument was programmed for denaturation at 95°C for 1 min, followed by 40 cycles of amplification at 95°C for 5 s, 57°C for 30 s, and 72°C for 30 s. The specific primers for the target genes and the internal reference gene (*EF-1α*) are given in **Supplementary Table S1**. Each sample was replicated three times and the data was analyzed using the 2^-ΔΔCt^ method (Livak and Schmittgen, 2001).

### Statistical Analysis

The data were analyzed using analysis of variance (ANOVA) followed by Duncan’s multiple-range test (*P* ≤ 0.05) using statistical software SPSS version 17.0 (SPSS Inc., Chicago, IL, USA). All experiments were repeated twice.

## Results

### VOCs Produced by SYST2 Enhance Plant Growth in Tomato

**FIGURE 1:**
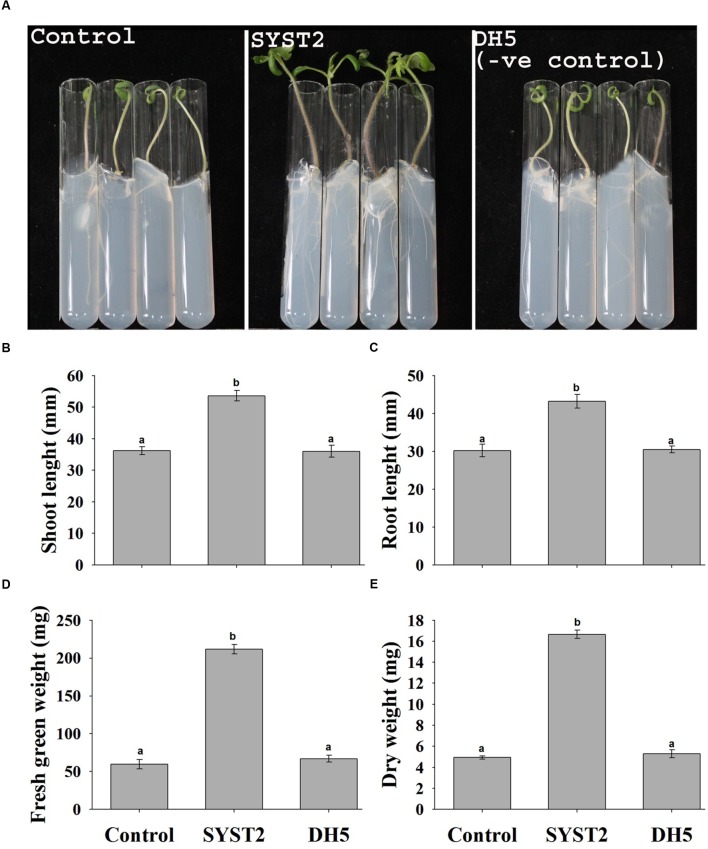
**Effects of SYST2 volatile organic compounds (VOCs) on plant growth enhancement *in vitro*.** Surface sterilized tomato seeds were placed individually in test tubes, and the resulting seedlings were grown in the presence of 20 μl *Bacillus subtilis* SYST2 culture (10^7^ CFU/ml) in sealed jars in an incubator for 14 days at 25°C. The effects of SYST2 VOCs on plant growth were observed visually **(A)** and by recording the differences in shoot length **(B)**, root length **(C)**, fresh green weight **(D)**, and dry weight **(E)** after 2 weeks of exposure. Error bars indicate the standard deviation of the mean (*n* = 5). Different lower case letters above the columns represent significant differences between treatments at *P* = 0.05. Experiments were repeated three times with similar results.

The plant growth promoting potential of VOCs produced by *B. subtilis* SYST2 was examined by growing plants in air-tight tissue culture tubes exposed to SYST2 VOCs for 14 days. A significant increase was observed in fresh green weight, dry weight, leaf area, and root and shoot length of tomato plants exposed to SYST2 VOCs as compared to the control. The VOCs emitted by SYST2 enhanced both the fresh and dry weights of tomato plants significantly, by 3.55- and 3.37-fold, respectively, compared to the controls, and increased shoot and root lengths by 1.48- and 1.43-fold, respectively. No significant differences were noted when the results of plants exposed to the VOCs of *E. coli* DH5α (negative control) were compared to the water control (**Figure 1**), confirming the plant growth promoting potential of VOCs produced by SYST2.

**FIGURE 2:**
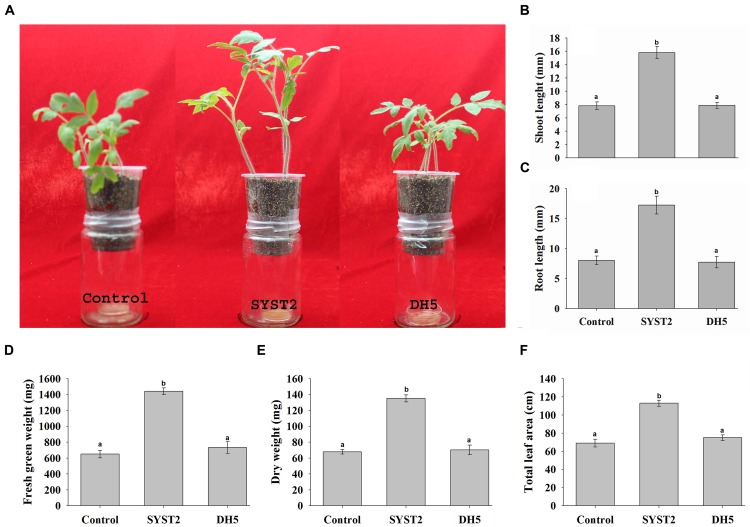
**Effects of SYST2 VOCs on plants grown in pots.** Three tomato seedlings of equal size were transferred to plastic pots containing soil, fixed on glass jars containing a culture of *B. subtilis* SYST2 at the bottom, and sealed with parafilm to avoid escape of the bacterial VOCs. Small (2 mm) holes in the bottom of the pots allowed for root exposure to VOCs. The pots in jars were incubated at 25°C under a 12 h light/12 h dark photoperiod for 30 days. Plant growth promoting activity was assessed visually **(A)**, and by differences in shoot length **(B)**, root length **(C)**, fresh green weight **(D)**, dry weight **(E)**, and total leaf area **(F)** between control plants and plants exposed to VOCs. Error bars indicate the standard deviation of the mean (*n* = 5). Letters above the columns represent significant differences between treatments at *P* = 0.05. Experiments were repeated in triplicate with similar results.

To investigate the plant growth promoting potential of VOCs at a broader level, we conducted pot experiments as described by Park et al. (2015). One-week-old tomato seedlings were transplanted into pots attached to the inside of jars containing a culture of SYST2 at the bottom. In this way, we tried to reproduce natural conditions in which volatile compounds can come into contact with tomato plants, similar to conditions in the soil. After a 4-week exposure, the analysis of the data revealed a significant enhancement in the growth of tomato seedlings in terms of fresh green and dry weight, root and shoot length, and leaf area as compared to the water and DH5α controls (**Figures 2A–F**). In the pot experiments, an almost twofold increase was observed for all parameters compared to the water control; fresh weight (2.21-fold), dry weight (1.98-fold), shoot length (2.02-fold), root length (2.05-fold), and leaf area (1.54-fold) (**Figures 2B–F**). Very similar results were found in comparisons to the DH5α control. Based on our data, we can state that the growth and development of tomato seedlings is stimulated by *B. subtilis* SYST2 VOCs both in test tube and pot experiments.

### Identification of VOCs Produced by SYST2

The growth promotion data indicated that VOCs produced by SYST2 might play key roles in stimulating plant growth and development. Therefore, we collected the VOCs produced by SYST2 by growing 20 μl cultures on MS medium in a 100 ml vial for 4 days at 28°C and analyzed them by SPME coupled with GC-MS. Mass spectra data of the volatile compounds were analyzed using the data in the NIST/EPA/NIH Mass Spectrum Library. Eleven volatile compounds which had relatively high peak areas, e.g., ≥1%, and were not similar to the control, were identified from SYST2. Retention times ranged from 2 to 15 min, with molecular weights ranging from 86 to 444 Da (**Table 1** and **Supplementary Figure S1**). The production of two identified VOCs albuterol and 1,3 propanediol were further confirmed and quantified by comparing with standard compounds (**Supplementary Figure S1**). The identified VOCs included four alcohols (albuterol, 3-Methyl-3-buten-1-ol, 2-butyl-1-octanol, and dimethyl silanediol), and two acids (methylphosphonic acid and benzoic acid). In addition, an ester (1,3-propanediol, 2-methyl-,dipropanoate); two ethers (octamethyl cyclotetrasiloxane and dodecamethyl cyclohexasiloxane), and two phenols (1,2-benzenediol,3,5-bis(1,1dimethylethyl), and 2-tert-butyl-4-methylphenol) were present in the samples collected from SYST2. The alcohol albuterol displayed the highest peak of the 11 compounds detected in the VOC spectrum emitted by SYST2 (**Supplementary Figure S1**).

**Table 1:** HS-SPME/gas chromatography-mass spectrometry (GC-MS) profile of volatile organic compounds (VOCs) produced by *Bacillus subtilis* SYST2.

RT (min:s)	Total area (%)	Possible compounds	CAS No.	Mol. weight	Effect on plant growth	
					
					100 ng/100 μl in 1 ml DMSO	200 ng/200 μl in 1 ml DMSO
2.46	4.293	Albuterol	18559-94-9	239	+Ve effect	+Ve effect
3.010	1.661	Methylphosphonic acid	993-13-5	96	NIL	NIL
3.131	2.896	3-Methyl-3-buten-1-ol	763-32-6	86	NIL	NIL
3.197	4.258	1,3-Propanediol, 2-methyl-,dipropanoate	54932-83-1	202	+Ve effect	+Ve effect
3.289	2.119	Silanediol, dimethyl	1066-42-8	92	NIL	NIL
6.392	3.03	1,2-Benzenediol,3,5-bis(1,1-dimethylethyl)	1020-31-1	222	NIL	NIL
9.387	3.249	Cyclotetrasiloxane, octamethyl-	556-67-2	296	NIL	-Ve effect
11.390	1.439	1-Octanol,2-butyl	3913-02-8	186	NIL	NIL
11.922	2.120	Benzoic acid		122	NIL	-Ve effect
14.399	1.61	Cyclohexasiloxane, dodecamethyl-	540-97-6	444	NIL	NIL
15.088	1.475	Phenol,2-(1,1-dimethylethyl)-4-methyl	2409-55-4	164	NIL	NIL


*Similar compounds present both in inoculated and non-inoculated MS medium were excluded. Several minor air-peaks (≤1% of the total area) were also not included. RT, retention time.*

### Albuterol and 1,3-Propanediol Are the VOCs That Possess Plant Growth Promoting Activity

**FIGURE 3:**
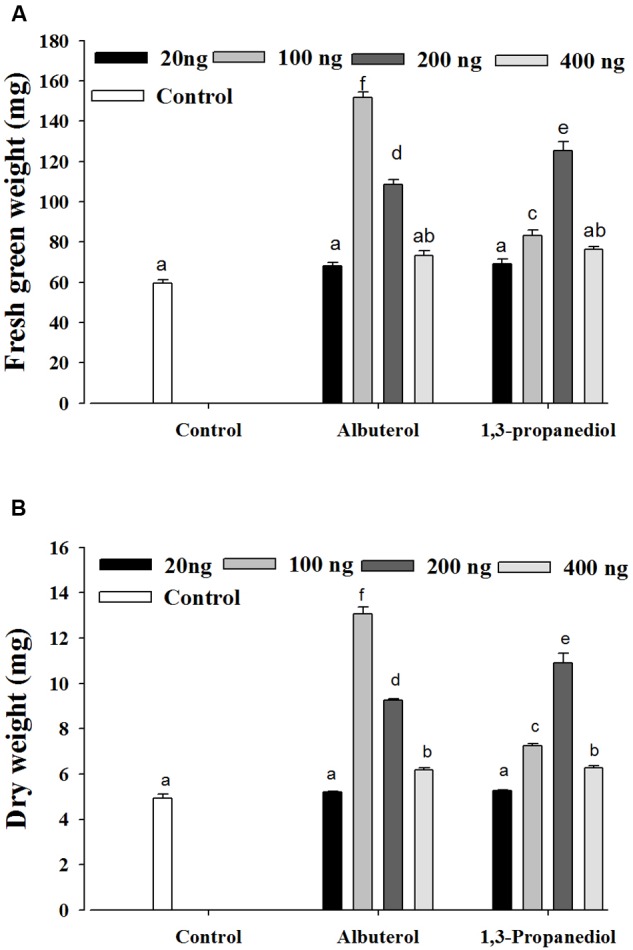
**Evaluation of individual VOCs produced by *B. subtilis* SYST2.** Tomato seedlings were exposed to the synthetic VOCs albuterol and 1,3-propanediol dissolved in DMSO at four different concentrations (20, 100, 200, and 400 ng) in test tubes in sealed jars for 14 days at 25°C under a 12 h light/12 h dark photoperiod. Plant fresh weights **(A)** and dry weights **(B)** were determined. Error bars indicate the standard deviation from the mean. Letters above the columns indicate a significant difference at *P* < 0.05. Experiments were replicated three times.

All of the compounds identified by SYST2 were evaluated individually for their plant growth promoting ability. Only two compounds, albuterol and 1,3-propanediol, showed positive effects with respect to stimulating plant growth promotion as compared to the control. Tomato seedlings were grown from surface-sterilized seeds in tissue culture test tubes inside air-tight tissue culture jars. The individual compounds were dissolved in DMSO to give final concentrations of 20, 100, 200, and 500 ng, applied to a Petri plate at the bottom of tissue culture jar, and incubated for 14 days at 25°C. The results showed that albuterol and 1,3-propanediol stimulated growth significantly at all doses except 20 ng relative to the control, but at varying levels. A significant difference was observed in fresh green weight, dry weight, and root and shoot lengths in tomato plants after exposure to these volatile compounds. 1,3-propanediol (200 ng) enhanced fresh green weight by 2.10-fold and dry weight by 2.20-fold, while albuterol (100 ng) increased fresh green weight by 2.54-fold and dry weight by 2.64-fold as compared to the control (**Figures 3A,B**).

### SYST2 VOCs Regulate Photosynthesis and Growth Hormones

**FIGURE 4:**
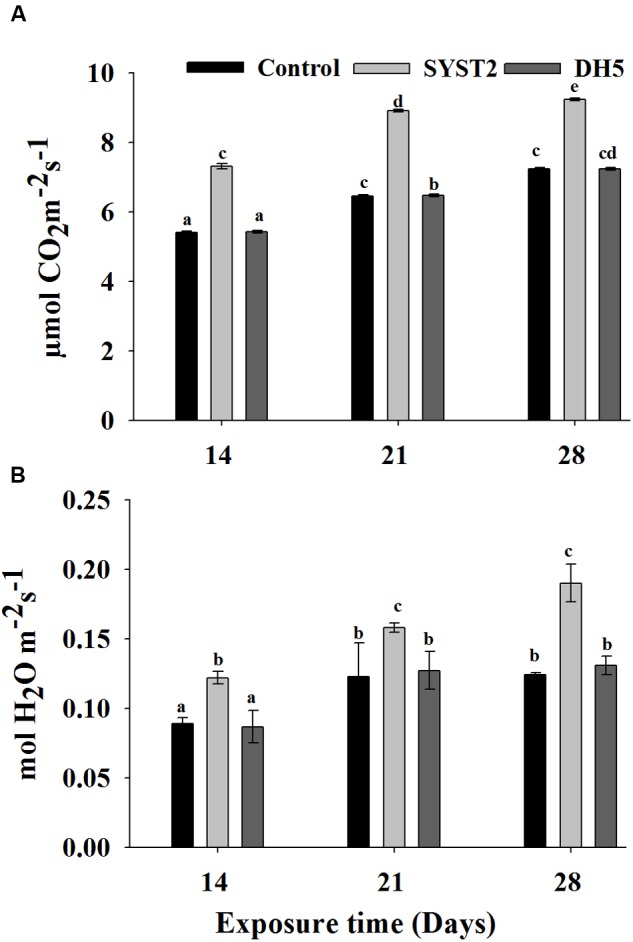
**Effects of SYST2 VOCs on photosynthesis rate and stomatal conductance.** Photosynthesis rates **(A)** and stomatal conductance **(B)** were measured after 14, 21, and 28 days of exposure to SYST2 VOCs from three randomly-selected leaves. Error bars represent standard deviations of the means. Lower case letters above the columns indicate a significant difference at *P* < 0.05. Experiments were conducted in triplicate.

Photosynthesis and stomatal conductance were measured after exposure to VOCs produced by SYST2 at 7-day intervals, beginning at 14 days and continuing until 28 days of incubation. The stomatal conductance and photosynthesis rate were significantly increased after exposure to VOCs as compared to the water and DH5α controls. Although the data presented in **Figure 4** demonstrated that there is no significant difference in stomatal conductance after 14 days and 28 days exposure but photosynthesis was clearly increased comparative to both water and DH5α control. Stomatal conductance was increased with increasing exposure time of plants to the VOCs produced by SYST2.

**FIGURE 5:**
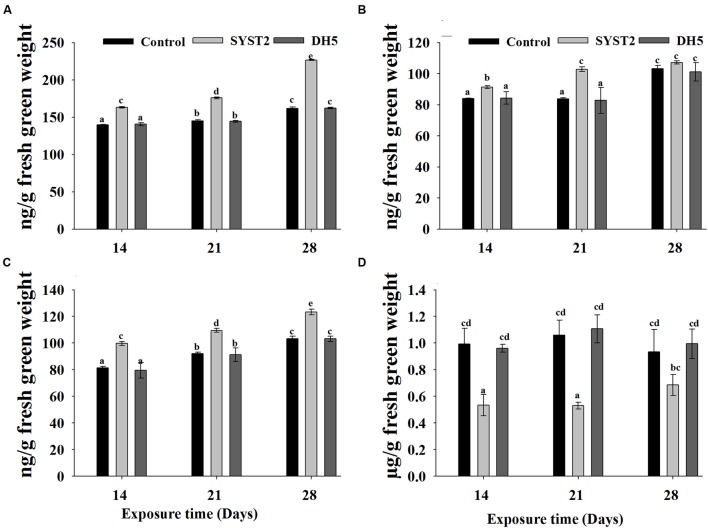
**Effect of SYST2-produced VOCs on plant growth hormones.** Plant samples were collected after 14, 21, and 28 days of exposure to VOCs produced by SYST2. The endogenous contents of phytohormones were determined based on the methods of Kelen et al. (2004) using UPLC; auxin **(A)**, gibberellin **(B)**, cytokinin **(C)**, and ethylene **(D)**. Error bars represent standard deviations of the means. Values are the means of three replications. Lower case letters above the columns indicate a significant difference at *P* < 0.05.

Although synthesis of plant growth hormones by PGPR as a growth promoting mechanism has been reported, the results of earlier studies are insufficient to conclude clearly whether there are interactions between VOCs produced by PGPR strains and the endogenous content of plant growth hormones. To demonstrate the effect of VOCs on the endogenous contents of auxin, cytokinin, and gibberellin, we quantified these hormones after exposure to SYST2-VOCs in newly growing leaves and roots of tomato seedlings. The auxin content clearly increased in SYST2-exposed plants compared to the control. Auxin accumulation reached the maximum at 28 days of exposure (1.4-fold), while it increased by 1.16-fold and 1.21-fold after 14 days and 21 days exposure, respectively, compared to both controls (**Figure 5A**). Similarly, cytokinin levels also increased upon exposure to VOCs but at lesser extent compared to auxin. Results showed an increase of 1.22-fold, 1.18-fold, and 1.19-fold at 14, 21, and 28 days of exposure to SYST2 VOCs compared to the control (**Figure 5C**). There were no significant differences in gibberellin contents after 28 days of exposure to VOCs compared to the control. A 1.2-fold increase was observed in endogenous GA content following exposure to SYST2 VOCs for 21 days (**Figure 5B**). Ethylene contents were also analyzed in tomato seedlings exposed to SYST2 VOCs using a plant ethylene enzyme-linked immunosorbent assay (ELISA) kit. We found a significant decrease in ethylene content following the exposure to VOCs produced by *B. subtilis* SYST2 compared to the control (**Figure 5D**).

### VOCs Affect the Transcription of Genes Involved in Phytohormone Biosynthesis and Metabolism

**FIGURE 6:**
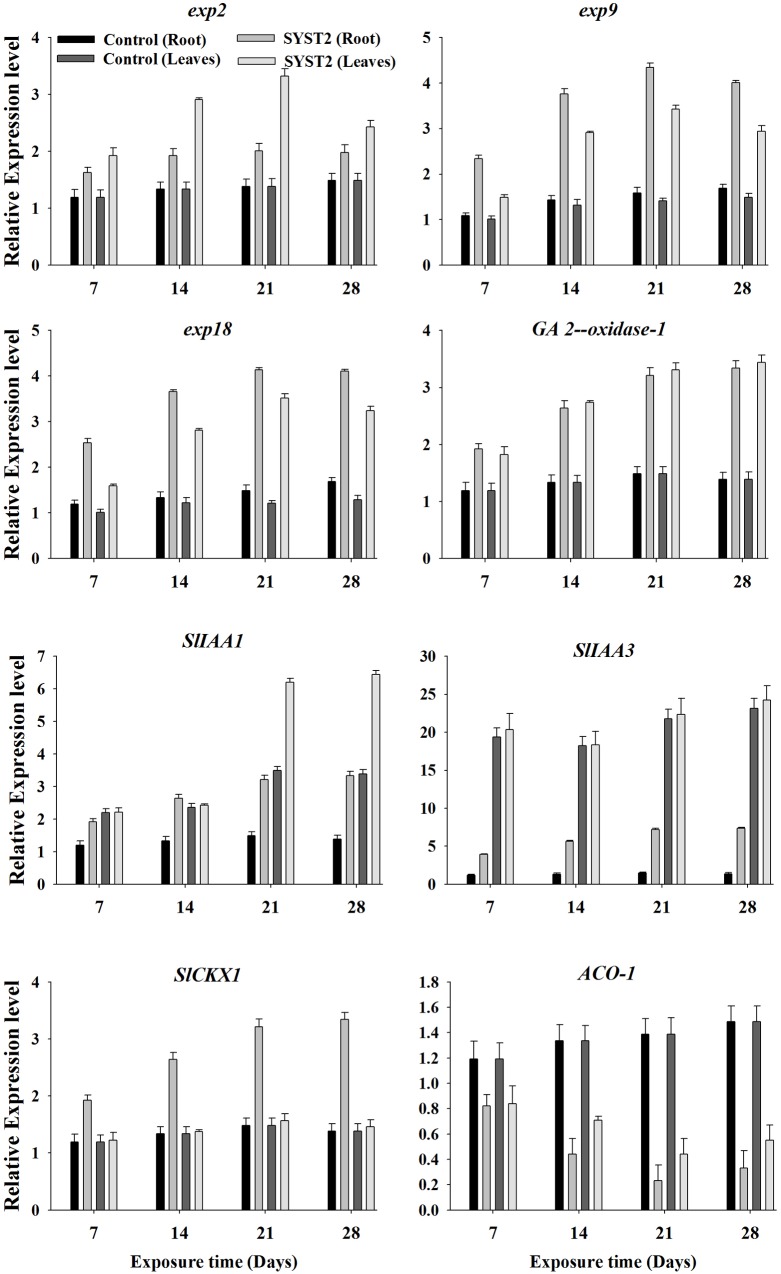
**Transcriptional expression profiles of genes involved in expansin, auxin, cytokinin, and gibberellin synthesis and metabolism.** qRT-PCR was performed with SYBR Green/Fluorescent qPCR master mix (Takara) on a Roche-480 system (Roche), using *EF-1α* as an internal reference. The relative expression levels of *Exp2. Exp9. Exp18. ACO-1*, GA (*20ox-1*), *SlIAA1. SlIAA3*, and *SlCKX1* were determined. Error bars indicate standard errors of the means. Different lower case letters above the error bars represent significant differences according to Duncan’s multiple-range test (*P* ≤ 0.05) using SPSS software (SPSS, Chicago, IL, USA).

To investigate whether the growth promoting activity of VOCs is related to changes in the relative expression of genes associated with expansin, IAA, GA, CK, and ethylene biosynthesis or metabolism, we examined the transcriptional levels of some key genes related to expansin (*Exp2. Exp9. Exp18*), ethylene (*ACO-1*), GA (20ox-1), auxin (*SlIAA1* and *SlIAA3*), and cytokinin (*SlCKX1*) using quantitative PCR (qRT-PCR). Newly-growing leaves and roots were sampled at 14, 21, and 28 days after exposure to VOCs produced by SYST2, total RNA was extracted, and the first-strand cDNA was synthesized from the polyadenylated mRNA. qRT-PCR assays performed with gene-specific primers showed that SYST2 VOCs altered the transcription of all the tested genes to varying degrees (**Figure 6**). The relative expression of the three expansin genes *Exp2. Exp9*, and *Exp18* was clearly increased after exposure to SYST2 VOCs in roots and young leaves. However, *Exp9* and *Exp18* showed higher levels of expression in the roots, while transcription of *Exp2* was more up-regulated in the leaves. Expression levels of *Exp2* and *Exp9* gradually increased and peaked after 21 days of exposure in both roots and young leaves, and *Exp18* showed no significant differences in expression after 21 and 28 days of exposure to SYST2 VOCs. A gradual but continuous increase in transcription was observed for the *20ox-1* (gibberellin synthesis) gene in leaves from 7 to 28 days of exposure, while no significant difference in expression was observed in roots. Our results showed that the highest relative up-regulation was observed for the expression of *SlIAA3* compared to *SlIAA1* and other genes involved in cytokinin or gibberellin synthesis. Similarly, SYST2 VOCs induced transcription of genes involved in cytokinin synthesis in roots. No significant effect was observed on the relative expression of *SlCKX1* in leaves exposed to VOCs. Our results showed a clear down-regulation of the *ACO-1* gene both in roots and leaves after exposure to SYST2 VOCs compared to the control. *ACO-1* encodes ACC oxidase, an enzyme that catalyzes the final step in ethylene biosynthesis.

## Discussion

Plants have the ability to detect and respond to microbial VOCs in their environment, which can result in plant growth regulation and induced systemic resistance. Our results clearly demonstrated the plant growth promoting activity of VOCs emitted by a new strain of *Bacillus subtilis*, SYST2 in tomato seedlings. Since 2003, the majority of studies on plant growth regulation by bacterial VOCs described the interaction of VOCs with plants *in vitro* (Ryu et al., 2003; Kwon et al., 2010; Lee et al., 2012; Kai and Piechulla, 2014; Xie et al., 2014). However, Park et al. (2015) recently reported that VOCs emitted by *P. fluorescens* SS101 can stimulate plant growth and development both *in vitro* and *in vivo* (Park et al., 2015), although the effect of VOCs on photosynthesis, phytohormones, and the expression of related genes is not clearly understood. Our results show that strain SYST2 produces VOCs that appear to increase the photosynthesis rate, regulate the endogenous contents of phytohormones, and alter the transcription of genes involved in gibberellin, auxin cytokinin, ethylene, and expansin synthesis, resulting in the stimulation of plant growth in tomato both *in vitro* and in pot experiments. A clear increase was observed in the fresh weight, dry weight, shoot length, root length, and leaf area of tomato plants following exposure to SYST2 VOCs and two synthetic compounds, albuterol and 1,3-propanediol, both in test tubes and in pot experiments. To test the effects of VOCs on plant growth promotion, most prior studies used the I-plate system because they were testing *Nicotiana* or *Arabidopsis* seeds. Because of the larger size of the tomato seeds and emerging seedlings, we modified the method, using glass test tubes in sealed tissue culture jars containing culture of *B. subtilis* SYST2 in a Petri plate at the bottom of jar. Our results showed a 3.5-fold increase in biomass of tomato seedlings after exposure to SYST2 VOCs, while a previous study showed an increase in biomass up to ninefold (Park et al., 2015). The proportionately smaller increase in biomass observed in our study could be due to the larger volume of the tissue culture jars and a lower concentration and availability of VOCs to the seedlings in the test tubes. Growth conditions in the jars and in the field are quite different in terms of growing media and the degree of interaction of the tomato seedlings with various environmental factors. Therefore, we shifted the experiment from *in vitro* conditions to pots in a growth chamber in order to evaluate the growth promoting activity in soil. Exposure of tomato plants to SYST2 VOCs under soil conditions also resulted in an increase in biomass, which agrees with the results of Park et al. (2015). PGPRs have been reported to modulate plant growth by producing auxin or other plant hormones (Macdonald et al., 1986; Timmusk et al., 1999), and by breaking down ethylene produced by plants (Glick et al., 1999); however, microarray results showed that microbial VOCs induced numerous physiological changes relating to growth hormones and photosynthesis (Zhang et al., 2007). Auxin and gibberellin are important for plant growth regulation, particularly cell elongation (Depuydt and Hardtke, 2011). Our results showed an increase in photosynthesis rate and auxin content, and differential expression of genes associated with auxin. VOCs produced by *B. subtilis* GBO3 have been shown to enhance photosynthetic activity by increasing chlorophyll content (Zhang et al., 2008; Xie et al., 2014) and by altering the expression of genes relating to chloroplasts, suggesting a significant relationship between the increase in photosynthetic rates and growth promotion (Zhang et al., 2008). Exposure to GB03 volatiles has been reported to induce endogenous auxin synthesis and regulate auxin transport in plants (Zhang et al., 2007). Our study showed that *SlIAA1* and *SlIAA3* were overexpressed in roots of VOC-treated plants. However, *SlIAA1* expression was relatively unaffected in leaves for the first 2 weeks and then increased dramatically at 21 and 28 days of exposure, while *SlIAA3* expression was almost unchanged in leaves. Our results are similar to those of Boerjan et al. (2003), who reported that higher auxin levels have a negative effect on leaf expansion; Arabidopsis mutants *sur1* and *sur2*, which overproduce auxin, show decreased leaf expansion (Boerjan et al., 2003). Similarly, IAA accumulation was reduced in leaves and enriched in root tips in response to the exposure to GBO3 VOCs, which in turn resulted in stimulation of lateral root formation (Zhang et al., 2007). In addition to auxin, numerous expansin genes have also been reported to be differentially expressed in response to exposure to VOCs. Expression of the expansin genes *EXPB1, EXPB3, EXP4*, and *EXP5* in *Arabidopsis* (Zhang et al., 2007), *EXP1, EXP2*, and *EXP6* in *Nicotiana* (Wang et al., 2009; Xie et al., 2014), and *EXPA5* in *Lactuca sativa* (Minerdi et al., 2009) was up-regulated, resulting in cell expansion. Similarly, our study showed that SYST2 VOCs caused differential expression of the expansin-related genes *Exp2, Exp9*, and also the gibberellin biosynthesis gene *20ox-1*. Exposure to SYST2 VOCs resulted in an increase in the endogenous level of cytokinin in roots, while no difference was observed in leaves. This increase may be due to increased transcription of the *SlCKX1* gene, which encodes cytokinin oxidase/dehydrogenase when exposed to VOCs. The involvement of the cytokinin pathway in growth regulation triggered by bacterial volatiles appears to be more important because the role of cytokinin in root development has been reported to be crucial (Bishopp et al., 2009). The Arabidopsis cytokinin mutants *cre1* and *ein2* have been reported to be insensitive to volatiles produced by *B. subtilis* GBO3, indicating a role for cytokinins in the perception of the PGPR signals (Ryu et al., 2003). GBO3 volatiles failed to elicit a biomass increase in *ein2* (ethylene insensitive2), while IN937a volatiles did not show growth promoting activity in the ethylene insensitive mutants *etr1* (ethylene response1), *ein2*, and *eir1* (ethylene insensitive root), suggesting that plants can modify their response to strain-specific signal transduction pathways (Ryu et al., 2004). Our results showed that VOCs produced by *B. subtilis* SYST2 decreased the ethylene content in tomato seedlings, which could be due to the fact that these VOCs also repressed transcriptional of the *ACO1* gene. These results support previously reported findings that ethylene biosynthesis (*ACO2. ACS4. ACS12*, and *SAM-2*) and ethylene response (*CHIB. ERF1*, and *GST1*) genes have been shown to respond to bacterial volatiles at the transcriptional level (Kwon et al., 2010).

## Conclusion

In conclusion, we observed that SYST2 VOCs possess significant plant growth promoting activity in tomato seedlings. Of 11 individual VOCs produced by this strain of *B. subtilis*, two, albuterol and 1,3-propanediol, were found to be the key growth promoting factors. Exposure to SYST2 VOCs induced differential expression of genes involved in expansin, auxin, gibberellin, cytokinin, and ethylene biosynthesis or metabolism, which was then reflected in the altered levels of the endogenous contents of the related hormones in roots and leaves, suggesting the involvement of these hormones in signal transduction pathways induced by volatiles. Our findings provide new insights into the interactions between SYST2 VOCs and tomato seedlings *in vitro* and in pot experiments in terms of phytohormones and the regulation of photosynthesis, resulting in the promotion of plant growth.

## Author Contributions

HT, QG, and XG conceived and designed the experiments. HT performed most of the experiments. MC and AH performed the quantitative real time-PCR and recorded photosynthesis rate in tomato, respectively. HW performed the phytohormone analysis. WR and LW analyzed the data. HT wrote the manuscript.

## Conflict of Interest Statement

The authors declare that the research was conducted in the absence of any commercial or financial relationships that could be construed as a potential conflict of interest.

## References

[B1] Bailly A. Weisskopf L. (2012). Current knowledge and future challenges. *Plant Signal. Behav.* 7 1–7. 10.4161/psb.7.1.18418 22301973 PMC3357376

[B2] Berberich T. Sugawara K. Harada M. Kusano T. (1995). Short communication molecular cloning, characterization and expression of an elongation factor 1 a gene in maize. *Plant Mol. Biol.* 45407 611–615. 10.1007/BF00020988 8534856

[B3] Bishopp A. Help H. Helariutta Y. (2009). “Chapter 1 cytokinin signaling during root development,” in *International Review of Cell and Molecular Biology*, ed. Kwang W. J. (San Diego, CA: Academic Press), 1–48. 10.1016/S1937-6448(09)76001-0 19584010

[B4] Boerjan W. Ralph J. Baucher M. (2003). Lignin biosynthesis. *Annu. Rev. Plant Biol.* 54 519–546. 10.1146/annurev.arplant.54.031902.134938 14503002

[B5] Depuydt S. Hardtke C. S. (2011). Hormone signalling crosstalk in plant growth regulation. *Curr. Biol.* 21 R365–R373. 10.1016/j.cub.2011.03.013 21549959

[B6] Farag M. A. Ryu C.-M. Sumner L. W. Paul W. P. (2006). GC-MS SPME profiling of rhizobacterial volatiles reveals prospective inducers of growth promotion and induced systemic resistance in plants. *Phytochemistry* 67 k2262–2268. 10.1016/j.phytochem.2006.07.021 16949113

[B7] Francis I. Holsters M. Vereecke D. (2010). The gram-positive side of plant-microbe interactions. *Environ. Microbiol.* 12 1–12. 10.1111/j.1462-2920.2009.01989.x 19624707

[B8] Glick B. R. (2005). Modulation of plant ethylene levels by the bacterial enzyme ACC deaminase. *FEMS Microbiol*. *Lett.* 251 1–7. 10.1016/j.femsle.2005.07.030 16099604

[B9] Glick B. R. Patten C. N. Holguin G. Penrose D. M. (1999). *Biochemical and Genetic Mechanisms Used by Plant Growth Promotion Bacteria.* London: Imperial College Press, 1–13.

[B10] Kai M. Piechulla B. (2014). Impact of volatiles of the rhizobacteria serratia odorifera on the moss physcomitrella patens. *Plant Signal. Behav.* 5 444–446. 10.4161/psb.5.4.11340 PMC7080412 20339320

[B11] Kelen M. Demiralay E. C. Sen S. Ozkan G. (2004). Separation of abscisic acid, indole-3-acetic acid, gibberellic acid in 99 R (*Vitis berlandieri* x *Vitis rupestris*) and rose oil (Rosa Damascena Mill.) by reversed phase liquid chromatography. *Turk. J. Chem.* 28 603–610.

[B12] Kim Y. C. Leveau J. Gardener B. B. M. Pierson E. A. Pierson L. S. Ryu C.-M. (2011). The multifactorial basis for plant health promotion by plant-associated bacteria. *Appl. Environ. Microbiol.* 77 1548–1555. 10.1128/AEM.01867-10 21216911 PMC3067257

[B13] Kwon Y. S. Ryu C.-M. Lee S. Park H. B. Han K. S. Lee J. H. (2010). Proteome analysis of *Arabidopsis* seedlings exposed to bacterial volatiles. *Planta* 1370 1355–1370. 10.1007/s00425-010-1259-x 20820802

[B14] Lee B. Farag M. A. Park H. B. Kloepper W. J. Lee S. H. Ryu C.-M. (2012). Induced resistance by a long-chain bacterial volatile: elicitation of plant systemic defense by a C13 volatile produced by *Paenibacillus polymyxa*. *PloS ONE* 7:e48744 10.1371/journal.pone.0048744 PMC3509098 23209558

[B15] Lemfack M. C. Nickel J. Dunkel M. Preissner R. Piechulla B. (2014). mVOC: adatabase of microbial volatiles. *Nucleic Acid Res.* 42 D744–D748. 10.1093/nar/gkt1250 24311565 PMC3964988

[B16] Livak K. J. Schmittgen T. D. (2001). Analysis of relative gene expression data using real-time quantitative PCR and the 2(-Delta Delta C(T)) Method. *Methods* 25 402–408. 10.1006/meth.2001.1262 11846609

[B17] Macdonald E. M. S. Powell G. K. Regier D. A. Glass N. L. Roberto F. Kosuge T. (1986). Secretion of zwatin, ribosylzeatin and ribosyl-1-methylzeatin by *Pseudomonas savastanoi* plasmid coded cytokinin biosynthesis. *Plant Physiol.* 82 742–747. 16665104 10.1104/pp.82.3.742 PMC1056201

[B18] Minerdi D. Bossi S. Gullino M. L. Garibaldi A. (2009). Volatile organic compounds: a potential direct long-distance mechanism for antagonistic action of *Fusarium oxysporum* strain MSA 35. *Environ. Microbiol.* 11 844–854. 10.1111/j.1462-2920.2008.01805.x 19396945

[B19] Murashige T. Skoog F. (1962). A revised medium for rapid growth and bioassays with tobacco tissue cultures. *Physiol. Plant.* 15 473–497. 10.1111/j.1399-3054.1962.tb08052.x

[B20] Ortíz-Castro R. Contreras-Cornejo H. A. Macías-Rodríguez L. López-Bucio J. (2014). The role of microbial signals in plant growth and development. *Plant Signal. Behav.* 4 701–712. 10.4161/psb.4.8.9047 PMC2801380 19820333

[B21] Park Y. S. Dutta S. Ann M. Raaijmakers J. M. Park K. (2015). Promotion of plant growth by *Pseudomonas fluorescens* strain SS101 via novel volatile organic compounds. *Biochem. Biophys. Res. Commun.* 461 361–365. 10.1016/j.bbrc.2015.04.039 25892516

[B22] Raza W. Yousaf S. Rajer F. U. (2016). Plant growth promoting activity of volatile organic compounds produced by biocontrol strains. *Sci. Lett.* 4 40–43.

[B23] Ryu C.-M. Farag M. A. Hu C.-H. Reddy M. S. Kloepper J. W. Pare P. W. (2004). Bacterial volatiles induce systemic resistance in *Arabidopsis*. *Plant Physiol.* 134 1017–1026. 10.1104/pp.103.026583 14976231 PMC389924

[B24] Ryu C.-M. Farag M. A. Hu C.-H. Reddy M. S. Wei H.-X. Pare P. W. (2003). Bacterial volatiles promote growth in *Arabidopsis*. *Proc. Natl. Acad. Sci. U.S.A.* 100 4927–4932. 10.1073/pnas.0730845100 12684534 PMC153657

[B25] Tahir H. A. S. Gu Q. Wu H. Niu Y. Huo R. Gao X. (2017). *Bacillus* volatiles adversely affect the physiology and ultra-structure of *Ralstonia solanacearum* and induce systemic resistance in tobacco against bacterial wilt. *Sci. Rep.* 7:40481 10.1038/srep40481 PMC5238454 28091587

[B26] Timmusk S. Nicander B. Granhall U. Tillberg E. (1999). Cytokinin production by *Paenibacillus polymyxa*. *Soil Biol. Biochem.* 31 1847–1852. 10.1007/s12088-009-0008-y

[B27] Wang S. Wu H. Qiao J. Ma L. Liu J. Xia Y. (2009). Molecular mechanism of plant growth promotion and induced systemic resistance to *Tobacco mosaic virus* by *Bacillus* Spp. *J. Microbiol. Biotechnol.* 19 1250–1258. 10.4014/jmb.0901.0008 19884788

[B28] Xie S. Wu H. Zang H. Wu L. Zhu Q. Gao X. (2014). Plant growth promotion by spermidine-producing *Bacillus subtilis* OKB105. *Mol. Plant Microbe Interact.* 27 655–663. 10.1094/MPMI-01-14-0010-R 24678831

[B29] Xie X. Zhang H. Pare P. (2009). Sustained growth promotion in *arabidopsis* with long-term exposure to the beneficial soil bacterium *Bacillus subtilis* (GB03). *Plant Signal. Behav.* 4 948–953. 10.4161/psb.4.10.9709 19826235 PMC2801358

[B30] Zhang H. Kim M. Krishnamachari V. Payton P. Sun Y. Grimson M. (2007). Rhizobacterial volatile emissions regulate auxin homeostasis and cell expansion in *Arabidopsis*. *Planta* 226 839–851. 10.1007/s00425-007-0530-2 17497164

[B31] Zhang H. Xie X. Kim M. S. Kornyeyev D. A. Holaday S. Paré P. W. (2008). Soil bacteria augment *Arabidopsis* photosynthesis by decreasing glucose sensing and abscisic acid levels in planta. *Plant J.* 56 264–273. 10.1111/j.1365-313X.2008.03593.x 18573192

[B32] Zou C. Li Z. Yu D. (2010). *Bacillus megaterium* strain XTBG34 promotes plant growth by producing 2-pentylfuran. *J. Microbiol.* 48 460–466. 10.1007/s12275-010-0068-z 20799087

