# Supplementary material for: Plant Growth Promotion by Volatile Organic Compounds Produced by Bacillus subtilis SYST2
Source: Front Microbiol. 2017 Feb 7;8:171. doi: 10.3389/fmicb.2017.00171 (PMC5293759; doi:10.3389/fmicb.2017.00171)
Supplement: Supplementary file 1 [file Data_Sheet_1.pdf]

Plant growth promotion by volatile organic compounds produced by *Bacillus subtilis* SYST2

Hafiz Abdul Samad Tahir[1+], Qin Gu[1+], Huijun Wu[1], Waseem Raza[2], Alwina Hanif[1], Liming Wu[1,] Massawe Venance Colman[1], Xuewen Gao[*]

[1]Department of Plant Pathology, College of Plant Protection, Nanjing Agricultural University, Key Laboratory of Integrated Management of Crop Diseases and Pests, Ministry of Education, Nanjing 210095, PR China

[2] Jiangsu Key Lab for Organic Solid Waste Utilization, Nanjing Agricultural University, Nanjing, China

[1+] Both authors contribute equally and share first authorship.

[*] Corresponding author: Xuewen Gao

Address: Nanjing Agricultural University, Weigang No.1, Nanjing 210095, PR China

Email: gaoxw@njau.edu.cn

Telephone/Fax: 86-25-8439526

SUPPLEMENTARY INFORMATION

Table S1: Primers used in the study

No	Oligo Name	Sequence $5^{/}$ to $3^{/}$
1	*EF-1α* (F)	AGACCACCAAGTACTACTGCAC
2	*EF-1α* (R)	CCACCAATCTTGTACACATCC
3	*Exp2* (F)	TGTTGGAGGTGCTGGTGATA
4	*Exp2* (R)	CCCCTTCAAAAGTTTGTCCA
5	*Exp9* (F)	GCTGGTATCGTCCCTGTTGT
6	*Exp 9 (*R)	CTGACCAACCAAAACGGAGT
7	*Exp18* (F)	TGGCAGAGTGCTCATGCTAC
8	*Exp18* (R)	ATGGCACCATTGTGGGTAGT
9	*20-x1* (F)	ACGAGAAGCCTTGTGCCTTA
10	*20-x1* (R)	AAAAGCGGCAACTCAAAGAA
11	*SlIAA1* (F)	TGGATGGTGCCCCTTATCTA
12	*SlIAA1* (R)	CCAAGGAACATCACCAACAA
13	*SlIAA3*(F)	GCATGGATGGAGCACCTTAT
14	*SlIAA3* (R)	ACATCACCAACAAGCATCCA
15	*SlCKX1* (F)	AACGATTGGTAGCGGACAAC
16	*SlCKX1* (R)	CTAACGCTCGCCTAATCACC
17	*ACO1* (F)	TGCGCCATCTTCCTACTTCT
18	*ACO1*(R)	CCTTGATCAAATCGGGCTTA

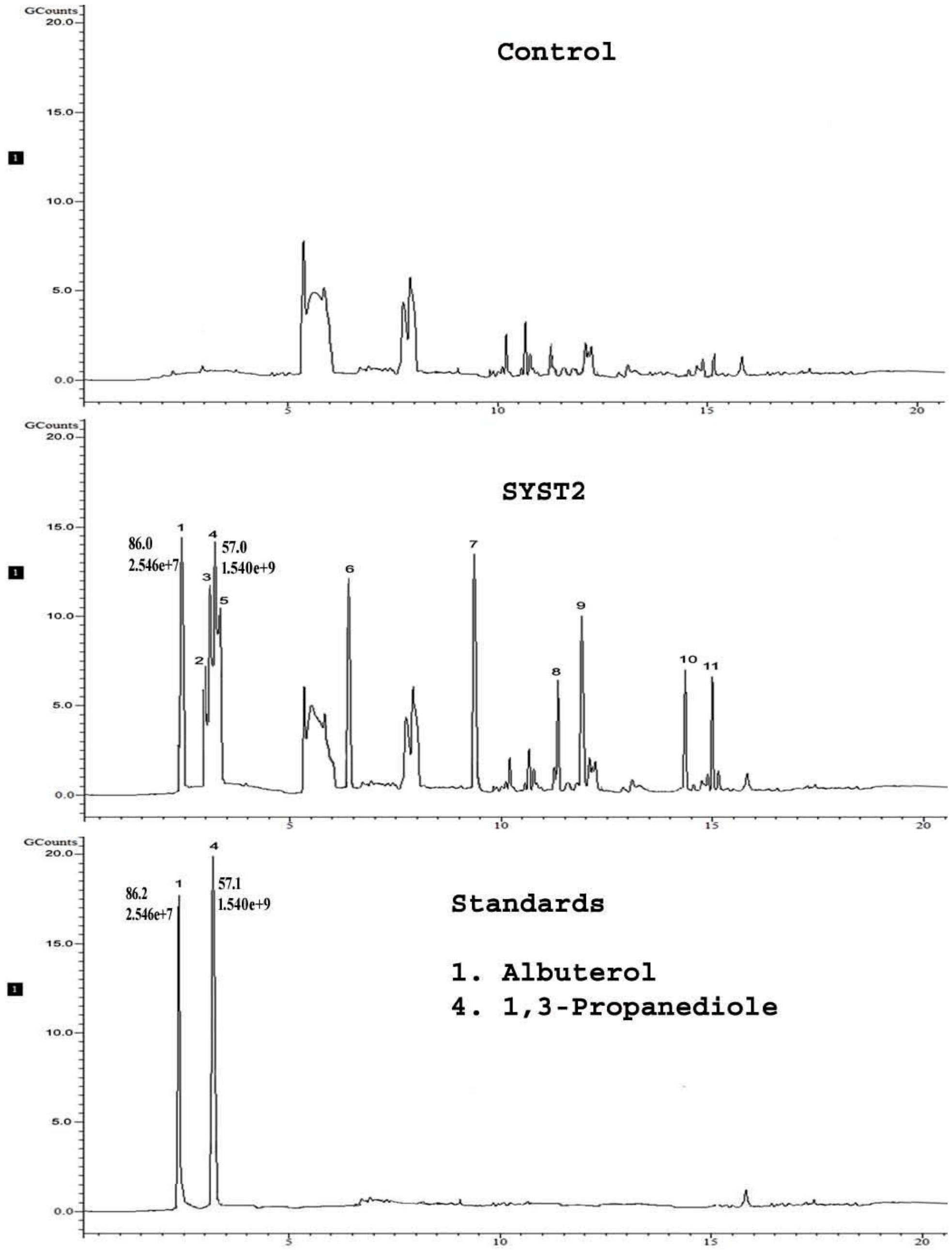

Figure S1: GC chromatograph of volatile organic compounds in control, produced by *Bacillus subtilis* SYST2 and two standard volatile organic compounds (albuterol and 1,3-propanediole). 1= Albuterol; 2 = Methylphosphonic acid; 3 = 3-Methyl-3-buten-1-ol; 4 = 1,3-Propanediol,2-methyl-,dipropanoate; 5 = Dimethyl silanediol,; 6 = 1,2-Benzenediol,3,5-bis(1,1-dimethylethyl); 7 = Cyclotetrasiloxane, octamethyl-; 8 = 1-Octanol,2-butyl; 9 = Benzoic acid; 10 = Cyclohexasiloxane, dodecamethyl-; 11= Phenol,2-(1,1-dimethylethyl)-4-methyl. M/Z value of compound 1 and 4 is given in graph.
